# Identification of Catechol as a New Marker for Detecting Propolis Adulteration

**DOI:** 10.3390/molecules190710208

**Published:** 2014-07-14

**Authors:** Shuai Huang, Cui-Ping Zhang, George Q. Li, Yue-Yi Sun, Kai Wang, Fu-Liang Hu

**Affiliations:** 1 College of Animal Sciences, Zhejiang University, Hangzhou 310058, China; E-Mails: asmallcaths@163.com (S.H.); lgzcplyx@aliyun.com (C.-P.Z.); 3110100394@zju.edu.cn (Y.-Y.S.); kaiwang628@gmail.com (K.W.); 2 Faculty of Pharmacy, University of Sydney, Sydney, NSW 2006, Australia; E-Mail: george.li@sydney.edu.au

**Keywords:** propolis, poplar extract, catechol, polyphenol oxidase, biosynthesis

## Abstract

Adulteration of propolis with poplar extract is a serious issue in the bee products market. The aim of this study was to identify marker compounds in adulterated propolis, and examine the transformation of chemical components from poplar buds to propolis. The chemical profiles of poplar extracts and propolis were compared, and a new marker compound, catechol, was isolated and identified from the extracts of poplar buds. The polyphenol oxidase, catechol oxidase, responsible for catalyzing oxidation of catechol was detected in poplar buds and propolis. The results indicate catechol can be used as a marker to detect propolis adulterated with poplar extract.

## 1. Introduction

The honeybee is a perennial species that exploits virtually all habitats on Earth, and their evolutionary and existent success are not only because of collecting nectar and pollen for fulfilling their nutritional needs, but also the ability to produce bee products: beeswax, venom, propolis and royal jelly [1,2]. As a case of ‘self-medication’ by the bee colonies, propolis has a role in the social immunity of honeybee, reducing the risk of disease and parasite transmission through the colony [3]. Since the ancient time, propolis has been used as a folk medicine for human health and preventing diseases, and it is gaining wider acceptance in popular medicine all over the world. A great number of studies have focused on the pharmacological and biological properties of propolis, including anti-inflammation [4], anticancer [5], antioxidative [6], immunomodulatory [7], antimicrobial, antibacterial, antiviral, and antifungal effects [7,8].

The materials available to bees for manufacturing propolis are plant buds, and substances actively secreted by plants as well as substances exuded from wounds in plants, but bees have a marked preference for one or a few sticky sources [3,9]. According to different geographical locations, poplar, conifer, birch, pine, alder, willow, palm, *Baccharis dracunculifolia* and *Dalbergia ecastaphyllum* are identified as the plant sources of propolis [10,11]. Among a multitude of botanical sources, *Populus* species are considered as the main plant origin of propolis all over the world, especially in the temperate zone [3]. Most propolis collected from Europe, North America, template Asia, has a similar chemical composition, color and smell as the extract of *Populus* bud. The yield of propolis is relatively low, and does not meet the demand of the growing market, leading to the adulteration of propolis with the extract of the poplar buds. This counterfeiting behavior has seriously disrupted the propolis industry, raising concerns on the quality, efficacy and safety of fake propolis.

A number of studies have been carried out to compare the chemical compositions of propolis and poplar extract. It has been shown that the flavonoids in these two natural products are very similar. Wu *et al.* found the differences between propolis and poplar extract are caused by the amounts of long-chain alkyl compounds [12]. Zhang *et al.* developed a HPLC method to use salicin to screen counterfeit propolis [13]. However, salicin is susceptible to acid and can be hydrolyzed to glucose and saligenin which are not detectable by the method. Therefore a new marker compound for distinguishing propolis from poplar extract is urgently needed.

The original plant resin can be modified by enzymes from honeybee [14], which leads to the differences of the chemical constitutes between the propolis and its botanical sources. For example, β-glucosidase has been purified from ventriculus, honey sac, and hypopharyngeal glands of *Apis mellifera* [15], and propolis. It hydrolyzes flavonoid mono-glucosides in plant resins during propolis collection and processing [16,17].

**Figure 1:**
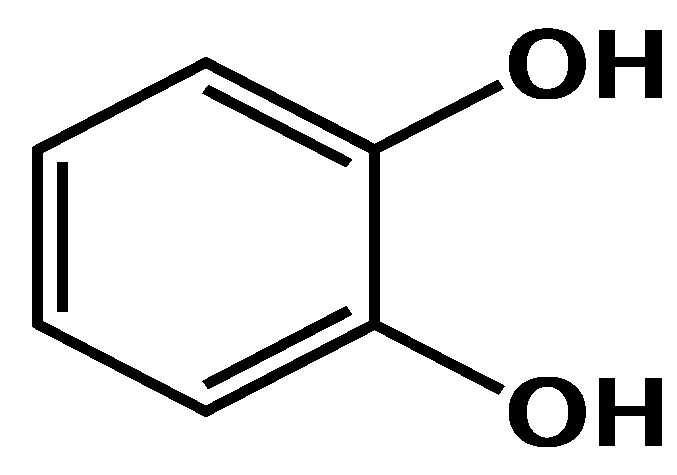
The chemical structure of catechol.

Catechol (*o*-diphenol, Figure 1) occurs naturally in fruits, vegetables and plants, along with polyphenol oxidase (PPO), an enzyme localized on the thylakoids of chloroplasts, in vesicles or other bodies of non-green plastid types [18]. PPO catalyzes two different reactions: hydroxylation of monophenols to *o*-diphenols and oxidation of *p*- and *o*-diphenols to *p*- and *o*-quinones [19]. The specific isozyme which works on *o*-diphenol substrates such as catechol is catechol oxidase (EC 1.10.3.1). Upon mixing polyphenol oxidase with the substrate in exposure to oxygen, the colorless catechol is oxidized to reddish-brown melanoid pigments *o*-diquinones, derivatives of benzoquinone [20].

**Figure 2:**
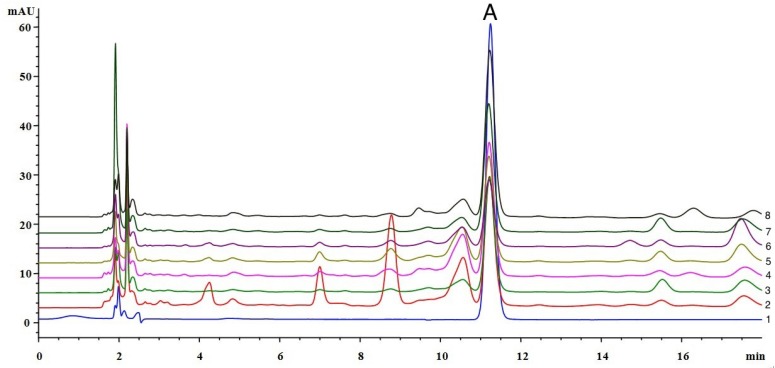
HPLC chromatogrsams of (1) catechol standard and (2–8) different poplar extracts.

In our previous studies, we reported that in addition to salicin, there was an unknown chemical component (shown as peak A in Figure 2) in poplar exact (gum) but not in propolis samples [13]. The aim of this study was to identify this marker compound to detect the adulteration of propolis. In addition, we also studied the presence of polyphenol oxidase, which may be a possible reason catechol is not present in propolis.

## 2. Results and Discussion

### 2.1. The Identification of Catechol in Poplar Extract

The unknown compound existing in the poplar extract was isolated as a colorless crystalline solid and had a molecular formula C_6_H_6_O_2_, as determined by the negative mode ESI-MS [*m/z* 109.1 (M−H)^−^]. According to the ^1^H-NMR, a typical AA'BB' coupling system signals at δ_H_ 6.75 (2H, m) and 6.65 (2H, m) were assigned to an *ortho*-disubstituded benzene ring. Correspondingly, the ^13^C-NMR showed three carbon signals at δ_C_ 146.3, 120.9, and 116.4, which indicated symmetry in the structure (Table 1). The negative mode ESI-MS, ^1^H-NMR, ^13^C-NMR profiles are listed in the Supplementary Information. The chemical data was consistent with an *ortho*-diphenol structure, catechol (Figure 1). Subsequently, the structure was confirmed by comparing with a catechol standard by HPLC (Figure 2).

**Table 1:** ^1^H-NMR and ^13^C-NMR data of catechol in CD_3_OD.

Atoms	^1^H-NMR	^13^C-NMR
1 and 2	-	146.3 (q)
3 and 6	6.65 (2H, dd, *J* = 3.8, 7.3 Hz)	120.9 (d)
4 and 5	6.75 (2H, dd, *J* = 3.8, 7.3 Hz)	116.4 (d)

Values are in (*δ*) ppm. Figures in parenthese are coupling constants (*J*) in Hz.

### 2.2. Determination of Catechol in Poplar Tree Extract and Propolis Samples

**Figure 3:**
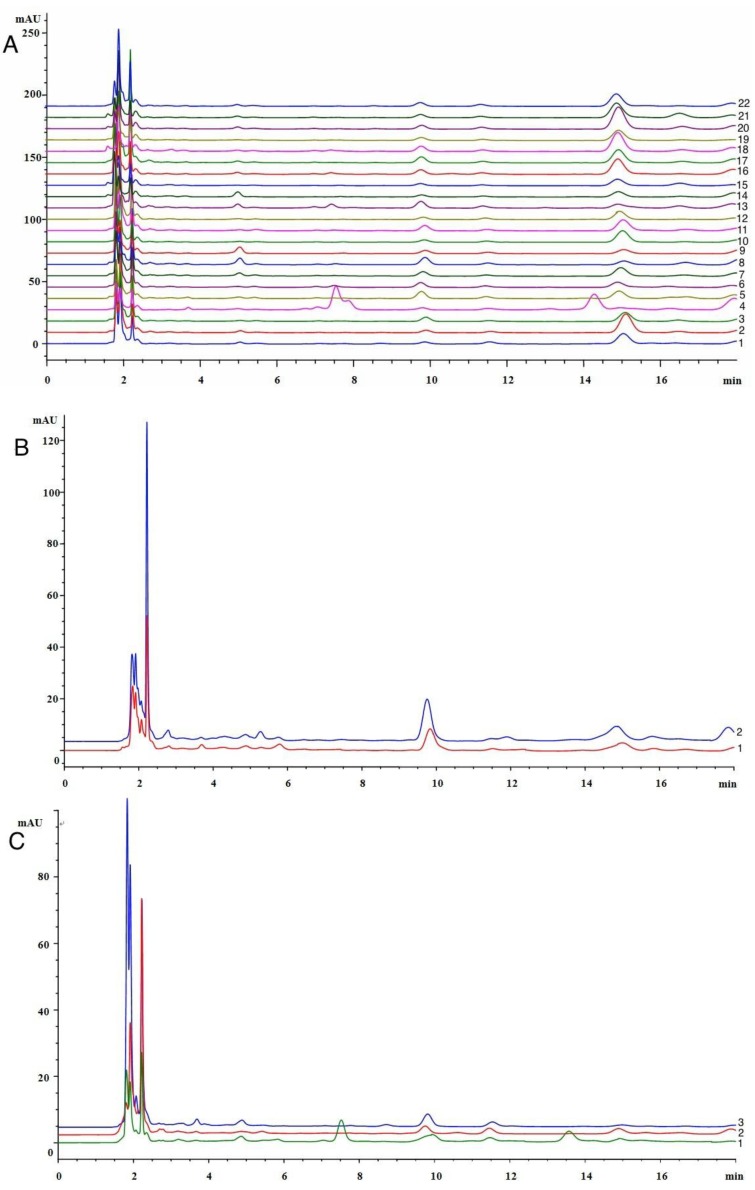
HPLC chromatograms of (**A**) 22 poplar type propolis; (**B**) two *Baccharis dracunculifolia* type proplis; (**C**) three *Eucalyptus* type propolis.

Catechol was detected in all poplar extract samples (Figure 2), and its content was relatively high, between 0.052 and 0.132 mg/g. However, catechol was undetectable in 22 Chinese propolis samples collected in different geographical locations and seasons, which were detected by a HPLC method and classed into poplar type propolis in our previous study [21] (Figure 3A). It was not detected in *Baccharis dracunculifolia* type (Figure 3B) and *Eucalyptus* type propolis either (Figure 3C). These results indicate that catechol is stable in the process of producing poplar extracts, and chemical analysis. These properties make catechol an ideal marker compound to detect propolis adulterated with poplar extract.

### 2.3. Detection the Polyphenol Oxidase (PPO) in Propolis and Poplar Buds

**Figure 4:**
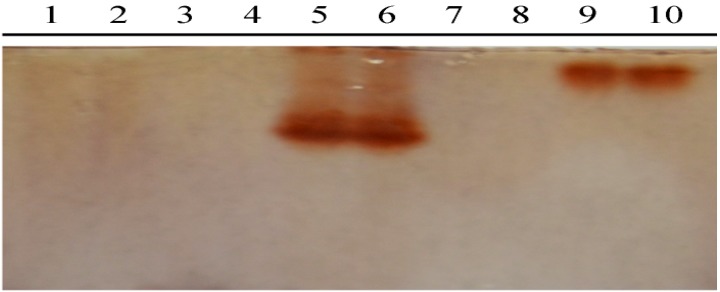
SDS-PAGE results of detecting polyphenol oxidase from protein extracts of poplar buds, poplar extracts, propolis, bee heads and bee bodies.

*Notes*: Lanes represent: 1–2, bees’ heads; 3–4, bees’ bodies; 5–6, propolis; 7–8, poplar tree gum; 9–10, poplar tree buds.

We hypothesized catechol from poplar buds is metabolized by enzymes in propolis, we therefore tested the existence of PPO in propolis, together with poplar buds, poplar extract, bee heads and bee bodies. Using catechol as a substrate, the SDS-PAGE gel with polyphenol oxidases turned reddish-brown after staining with the PPO chromogenic reagent. The SDS-PAGE gel (Figure 4), showed brown bands in the lanes of propolis and poplar buds, but not in poplar extracts, bee heads and bee bodies, indicating that PPO existed in the propolis and poplar buds, but not in the poplar extracts, bee heads and bee bodies. However, based on the different band position, the two PPOs in propolis and poplar buds were clearly recognized as isozymes with different molecular mass: the molecular mass of PPO in poplar buds was higher than that in propolis.

Polyphenol oxidase was identified from propolis for the first time when it has been purified from black poplar leaves [22] and its activity has been determined in poplar seedlings [23]. The enzyme might have been made inactive when alcohols was added [20], or by heating during the decoction process. Furthermore, the enzyme was not detected in either bee heads or bee bodies, indicating the origin of polyphenol oxidase in propolis is likely its raw material poplar buds, or other plants. However, the PPOs that exist in propolis and poplar buds are isozymes with different molecular masses, as the PPOs protein maybe hydrolyzed by some proteases existing in honeybee bodies [24] and digestive enzymes secreted from honeybee glands such as the salivary [25], or hypopharyngeal gland [26], without changing its activity. There is considerable evidence that PPO exists in a latent form in rudimentary thylakoids of leucoplasts, proplastids, or amyloplasts in healthy green tissues [20]. The vast majority of phenolic compounds in higher plant cells are located in the vacuole—A cellular location isolated from PPO. PPO is apparently not involved in biosynthesis of phenolics, but is involved with the production of *o*-quinones during pathogen invasion because it normally functions as a phenol oxidase playing a key role in plant defense only *in vivo* in senescent or damaged cells [18,27]. Honeybees collect plant tissues by beaks and chew them to produce propolis, during the process the cells are destroyed and PPO is activated, so that catechol could be oxidized into quinone in the propolis. In addition, potential natural *o*-diphenol substrates for PPO enzymes include caffeic acid derivatives and flavonoids, which can be transformed during the propolis collecting and producing process [19]. These results indicate that honeybees play an indispensable role in the process of propolis production, by adding their enzymes to modify the chemical compositions in plants, and also activating the enzymes in plant issues. However, literature about how honeybees transform chemical components of plant to produce propolis is still very limited, and further studies on the involvement of enzymes from plants and honeybees are warrant.

## 3. Experimental Section

### 3.1. Chemicals and Reagents

HPLC-grade methanol and acetonitrile were purchased from Merck (Darmstadt, Germany). Ethanol, petroleum ether, chloroform, ethyl acetate, phosphoric acid, acetic acid were analytical grade and purchased from Chemical Reagent Factory of Zhejiang University (Hangzhou, China). Ultra-pure water was purified by a Yjd-upws Ultra-Pure water system (Shanghai, China). Catechol was from Wiley Subscription Services, Inc. (San Francisco, CA, USA). 2% H_2_O_2_ and *para*-phenylenediamine were purchased from Yichen Shiye Corporation (Shanghai, China). Sephadex LH-20 was purchased from the Chemical Faculty of Zhejiang University (Hangzhou, China). All reagents used for HPLC analysis were filtered and degassed prior to use.

### 3.2. Materials

The buds of *Populus × canadensis* were collected from Zhejiang, China, in April 2013. Twenty-two poplar type propolis samples were scraped from the frame of the different beehives from different geographical locations of China from May to November 2013. Detailed sample information is presented in Table 2. Three *Eucalyptus* type propolis samples were obtained from Jims’ Bee Products (Young, Australia). Two *Baccharis* *dracunculifolia* type propolis samples were from Fengnaibao Company (Nanjing, China). The honeybees for detecting polyphenol oxidase were sampled from the beekeeping factory of Zhejiang University in September 2013. All samples were kept at −80 °C until analyzed.

**Table 2:** The geographical origins and collection time of poplar type propolis.

No.	Geographical Origin	Date of Collection
1	Shangshui, Henan	August 2013
2	Qiuxian, Hebei	May 2013
3	Shijiazhuang, Hebei	June 2013
4	Yicheng, Hubei	June 2013
5	Laodongkou, Hubei	June 2013
6	Meishan, Sichuan	July 2013
7	Wusong, Jilin	August 2013
8	Baishan, Jilin	August 2013
9	Ji’an, Jilin	July 2013
10	Dashiqiao, Liaoning	July 2013
11	Zhuanghe, Liaoning	July 2013
12	Faku, Liaoning	July 2013
13	Kongliu, Xinjiang	August 2013
14	Yilan, Heilongjiang	May 2013
15	Shuangyashan, Heilongjiang	August 2013
16	Fuyang, Anhui	June 2013
17	Huaibei, Anhui	June 2013
18	Tongcheng, Anhui	August 2013
19	Beijing	July 2013
20	Penglai, Shandong	May 2013
21	Longkou, Shandong	July 2013
22	Dong’e, Shandong	August 2013

### 3.3. Extraction and Isolation

Fresh buds of *Populus canadensis* were lyophilized at freeze dryer (Freeze dryer system 7960032, Labconco, Kansas, KA, USA) for three days before being ground. Around 300 g of the powder was extracted with 500 mL of pure ethanol by an ultrasonic cleaner for 1 h (SK20GT, Shanghai Kedao Ultrasonic Instrument Corporation, Shanghai, China). Then the extracting solution was filtered and concentrated to remove the ethanol in a rotary evaporator (RE-2000A, Shanghai Yarong Biochemistry Instrument Factory, Shanghai, China) at 35 °C until the volume of the extract solution reached less than 100 mL. Ultra-pure water (300 mL) was added to the ethanol extract solution and filtered. The water extract solution was condensed in a rotary evaporator at 35 °C, and then partitioned with petroleum ether, chloroform, and ethyl acetate, successively. The ethyl acetate fraction was subjected to Sephadex LH-20 gel column chromatography with 3,000 mL of ethanol to yield 20 fractions. The 10th fraction was separated by HPLC twice to yield catechol. The HPLC was an Agilent 1200 series (Agilent Technologies, Santa Clara, CA, USA), equipped with a G 1322 A vacuum degasser, a G1311A quaternary pump, a G1314B autosampler, a G1316A thermostatted column compartment, and a Sepax HP-C18 column (150 × 4.6 mm, 5 μm). The solvent was acetonitrile and 0.5% aqueous phosphoric acid (V/V) = 5:95; and the detector wavelength was 213 nm. The eluted fraction at *t_R_* = 11.5 min was further purified on HPLC under the same conditions.

### 3.4. MS and NMR

The identification of the purified catechol was carried out by MS, ^1^H- and ^13^C-NMR spectroscopy. HPLC-ESIMS analysis was carried out on an Agilent 6460 Triple Quad LC/MS fitted with an ESI source. The NMR spectra were performed with tetramethylsilane (TMS) as internal standard and CD_3_OD as solvent using a Bruker AVANCE III 600 MHz NMR spectrometer (Bruker, Stuttgart, Germany) with a standard broadband 5 mm BBFO probe.

### 3.5. Application of Catechol to Distinguish Poplar Extract and Propolis

Twenty-two poplar type propolis samples, three *Eucalyptus* type propolis samples, two *Baccharis* *dracunculifolia* type propolis, and seven poplar extract samples and the catechol standard were analyzed by the HPLC method described previously [13].

### 3.6. Polyphenol Oxidase (PPO) Preparation and 1-D SDS-PAGE

Propolis (1 g), poplar buds (1 g), bee heads (20), bee bodies (20) were extracted respectively with Tris buffer (3 mL, 0.1 M, pH 8.3), and ground in a mortar. The crude extract solutions were centrifuged at 12,000 ×*g* for 20 min at 4 °C, the supernatant were then assayed for PPO. Five μL of crude enzyme extract solution was used per lane for the SDS-PAGE (a 5% stacking gel overlying a 12% resolving gel) with two replicates for each sample. The SDS-PAGE gel was stained in the PPO chromogenic reagent A (2 g *para*-phenylenediamine in 18 mL acetic acid) and the PPO chromogenic reagent B (60 mL ultra-pure water, 1.5 mL 1% catechol and 0.3 mL 2% H_2_O_2_) for 3 min.

## 4. Conclusions

Catechol is a major marker compound in poplar extract that is used in the adulteration of propolis, and it can be used to detect propolis adulterated with poplar extract. The HPLC analytical process is simple and efficient for application by researchers and industry, providing a new method for authentication of propolis. Our results also suggest that catechol is likely oxidized by polyphenol oxidase, therefore absent in propolis.

## Supplementary Materials

Supplementary File 1
