# Supplementary material for: Identification of Catechol as a New Marker for Detecting Propolis Adulteration
Source: Molecules. 2014 Jul 14;19(7):10208–17. doi: 10.3390/molecules190710208 (PMC6271646; doi:10.3390/molecules190710208)

Supplementary File

Figure S1. The negative ions ESI-MS profile of catechol.

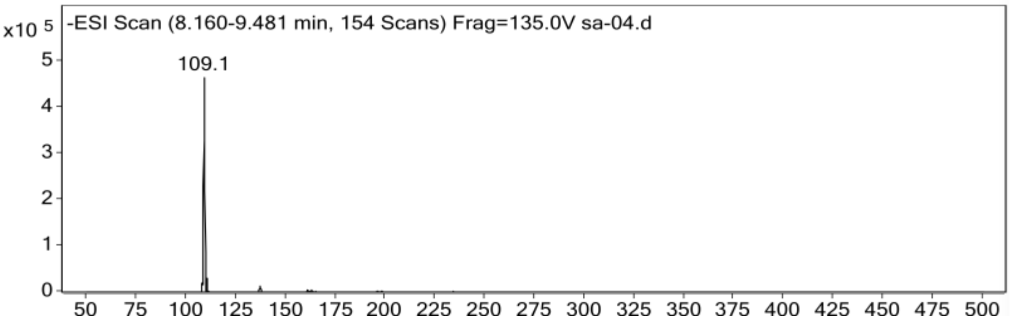

Figure S2. The ^{1}H-NMR profile of catechol (The original profile).

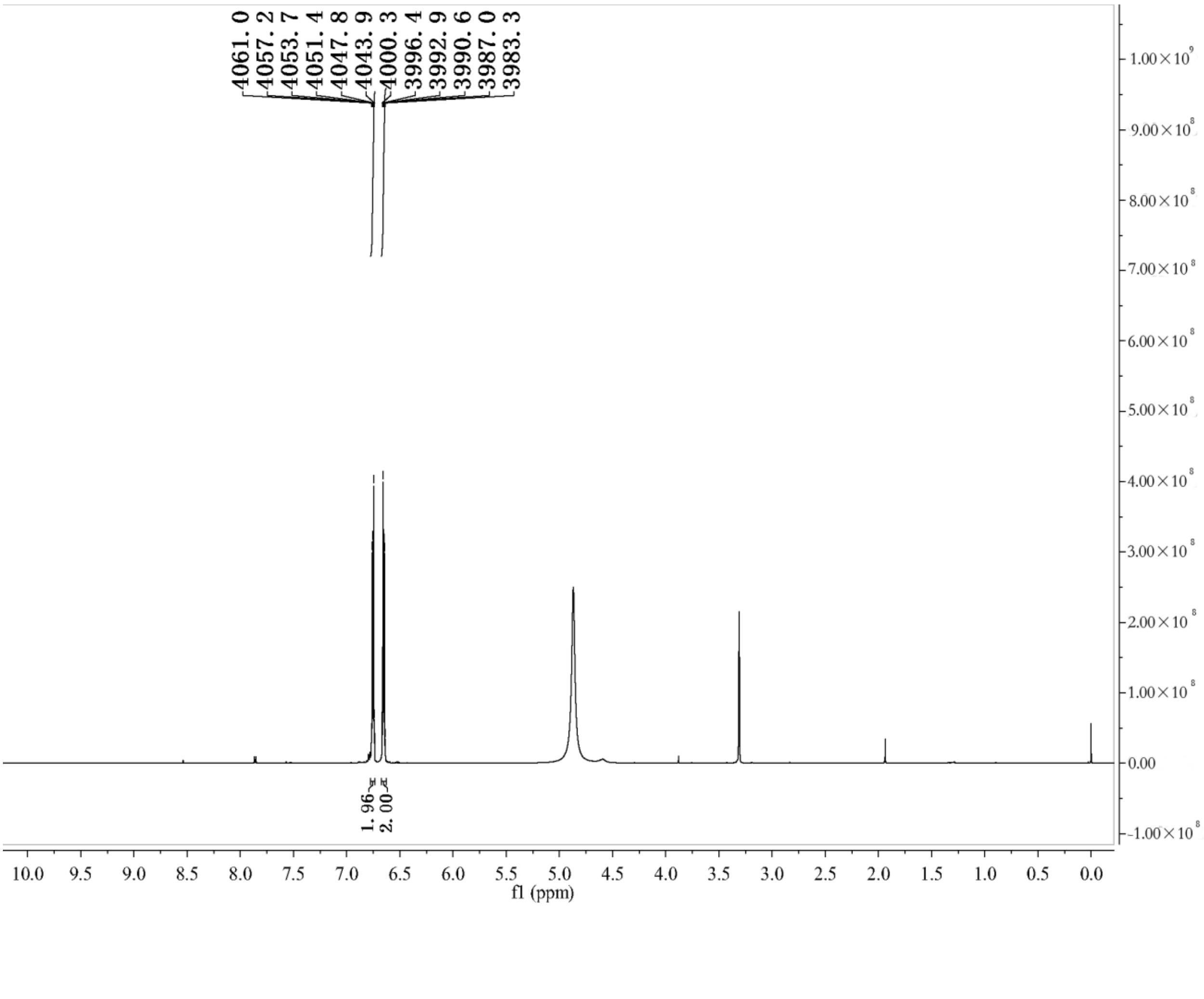

Figure S3. The ^{1}H-NMR profile of catechol (The enlarged profile of Figure S2).

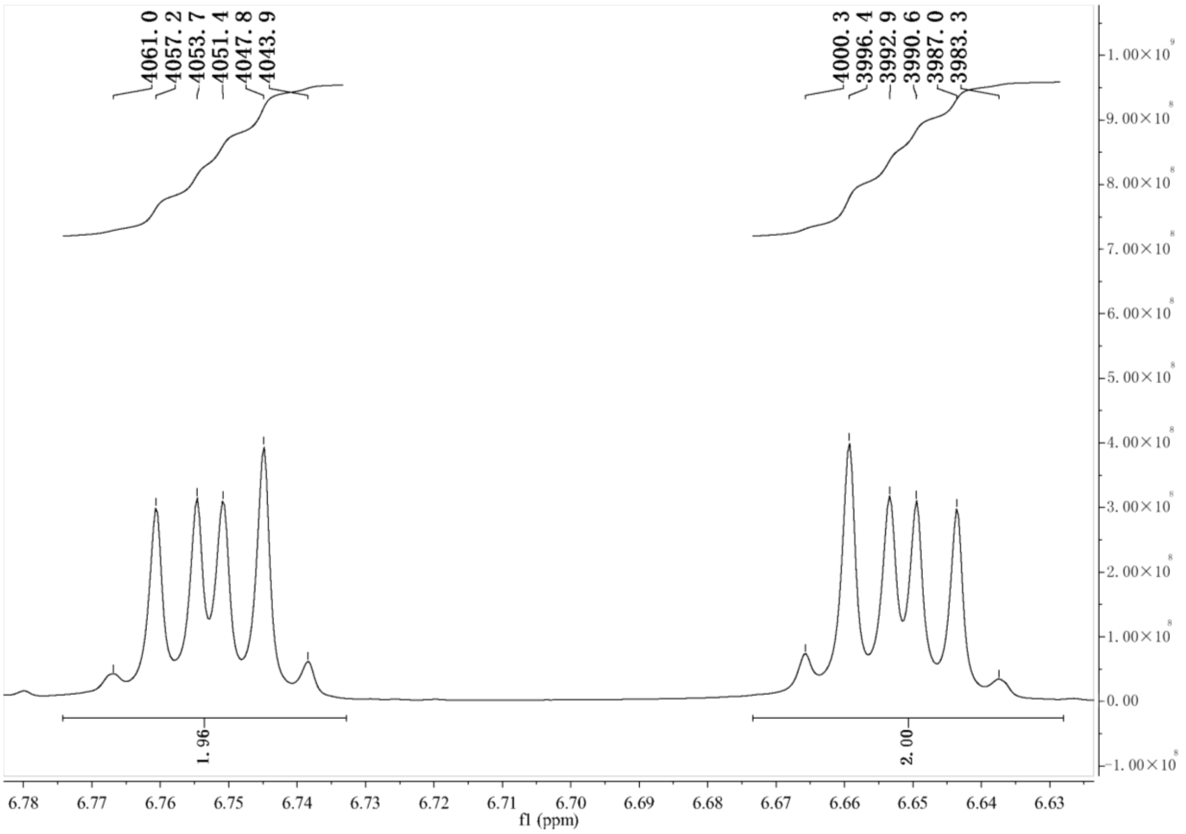

Figure S4. The ^{13}C-NMR profile of catechol.

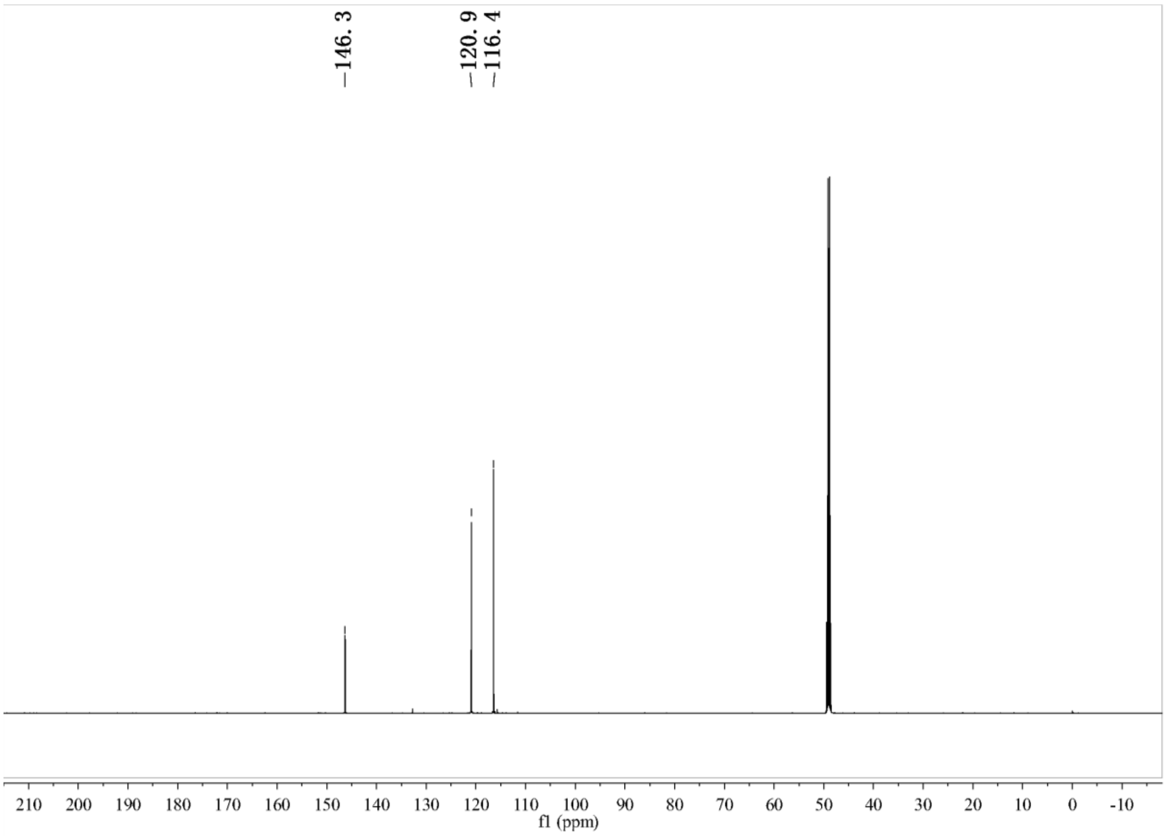

Supplement: Supplementary File 1 [file molecules-19-10208-s001.pdf]
